# Understanding primary care provider perspectives of the implementation of an integrated diabetes and mental health care solution

**DOI:** 10.1017/S1463423625100236

**Published:** 2025-07-18

**Authors:** Carly Whitmore, Janice Forsythe, Alegria Benzaquen, Michelle Domjancic, Osnat C. Melamed, Peter Selby, Diana Sherifali

**Affiliations:** 1 School of Nursing, McMaster University, Hamilton, ON, Canada; 2 INTREPID Lab, Centre for Addiction and Mental Health, Toronto, ON, Canada; 3 Diabetes Action Canada, Toronto, ON, Canada; 4 Department of Family and Community Medicine, University of Toronto, Toronto, ON, Canada; 5 Population Health Research Institute, Hamilton Health Sciences, Hamilton, ON, Canada

**Keywords:** Community care, cross-sectional survey, Diabetes, integrated care, mental health, primary care

## Abstract

**Aim:**

This research aimed to explore the perspectives of primary and community care providers on the challenges that hinder the delivery and uptake of personalized type 2 diabetes (T2D) care, with a focus on the integration of mental health support and care.

**Background:**

The day-to-day burden and demand of self-managing T2D can negatively impact quality of life and take a toll on mental health and psychological well-being. As a result, there is a need for personalized T2D self-management education and support that integrates mental health care. Despite the need for this personalized care, existing systems remain siloed, hindering access and uptake. In response, innovative, comprehensive, and collaborative models of care have been developed to address fragmentations in care. As individuals living with T2D often receive their care in primary care settings, linking mental health care to existing teams and networks in primary care settings is required. However, there is a need to understand how best to support access, adoption, and engagement with these models in these unique contexts.

**Methods:**

A cross-sectional survey was distributed to primary and community providers of an Ontario-based smoking cessation network. Survey data were analyzed descriptively with free text responses thematically reported.

**Findings:**

Survey respondents (n = 85) represented a broad mix of health professions across primary and community care settings. Addressing challenges to the delivery and uptake of personalized T2D care requires comprehensive strategies to address patient-, practice-, and system-level challenges. Findings from this survey identify the need to tailor these models of care to individual needs, clearly addressing mental health needs, and building strong partnership as means of enhancing accessibility and sustainability of integrated care delivery in primary care settings.

## Background

Living with type 2 diabetes (T2D) is not easy as the physical demands and chronicity of the condition necessitate constant and complex monitoring, management, and decision making (Robinson *et al.*, 2023). The day-to-day burden and demand of self-managing T2D can negatively impact quality of life and often takes a toll on mental health and psychological well-being, leading to distress, stress, and symptoms of anxiety, depression, and disordered eating (Diabetes Canada, 2018, Centers for Disease Control and Prevention, 2023, Egede *et al.*, 2005). Among adults living with T2D, the prevalence of co-occurring mental illness such as depression can be as high as 34% for women and 23% for men - twofold higher than in the general population (Khaledi *et al.*, 2019, Melamed *et al.*, 2022), while diabetes distress, described as the psychological consequences of living and managing diabetes (Polonsky *et al.*, 2005, Fisher *et al.*, 2012), is even more common, with an overall presence of 36% (Perrin *et al.*, 2017). For those living with T2D, poor mental health leads to an increase in primary care and emergency room visits that drive up healthcare costs by 70% compared to those without (Robinson *et al.*, 2023). These demands, overlaid with compounded stigma and shame can contribute to burn-out and disengagement from care and self-management (Abdoli *et al.*, 2021). This psychological ill-being ultimately drives a complex tension between glycemic control and mental health, and these adverse mental health states are more common amongst those who experience social, structural, and economic disadvantages living with diabetes, compared to individuals and communities who do not experience this degree of marginalization (Fazli and GL., 2023). Occurring in the context of the growing rates of obesity, sedentary lifestyles, and a socioculturally diverse and aging population, the increasing prevalence of T2D and concurrent mental health challenges is an urgent health problem worldwide.

Despite the increasing prevalence of these co-occurring conditions, related physical and mental health systems and care approaches remain siloed. As a result of the increasing complexity associated with living with T2D, the need for personalized T2D self-management education, support, and culturally safe and responsive care is also rising. This includes T2D treatment and management plans that are tailored to consider each individual and their specific health goals, lifestyle, and preferences (Williams *et al.*, 2022). Historically, where such programs are available, challenges regarding geography, finances, and long wait times create and enhance barriers to uptake and engagement. Consequently, those individuals who require this level of integrated care, including equity-deserving groups, older adults, and newcomers and immigrants, do not receive it. As a result, rather than receiving care in appropriate settings such as primary care, these individuals have become some of the highest users of an increasingly strained acute care healthcare system (Hutter *et al.*, 2010).

In response, innovative, comprehensive, and collaborative models of care have been developed to address fragmentations in care (Racey *et al.*, 2023). As individuals living with T2D often receive care in primary care settings, linking these programs to existing care teams and networks, and supplementing care already provided is a crucial piece to this puzzle. Successful delivery of integrated diabetes care in primary care settings poses many challenges. Known barriers such as communication issues, including the interoperability of electronic health records technology, and constraints on time and resources, must be considered. One example of such an innovation is the Technology-Enabled Collaborative Care for type 2 Diabetes and Mental Health (TECC-DM) model, a co-designed intervention aimed at delivering integrated diabetes and mental health care using virtual technologies.

By mobilizing findings from a successful feasibility study, there is an opportunity to further understand how to best support access, adoption, and engagement in an innovative and integrated virtual care model for individuals living with T2D and mental health challenges. To understand the purpose of this research, it is essential to first describe the work to date and context within which this research was conducted.

**Overview of the Research Context – the Technology-Enabled Collaborative Care for type 2 Diabetes and Mental health (TECC-DM) Model of Care**. A co-designed (people with lived experience, end-users, healthcare providers) mixed methods sequential explanatory feasibility study was completed in June 2022 using common, widely available and omnichannel technologies (e.g., REDCap, Webex) (Voljtila *et al.*, 2023). This project aimed to enhance self-care and self-efficacy by addressing attitudes, knowledge, and behaviour through virtual collaborative care, including health coaching, care coordination, and integrated diabetes and mental health care. Moving beyond what had traditionally been delivered as siloed care, the TECC-DM project evaluated an innovative integrated care model (Sherifali *et al.*, 2024). Thirty-one participants living with T2D and mental health challenges (e.g., depression, anxiety, and diabetes distress), received the TECC-DM intervention over 8-12 weeks. This included weekly one-on-one coaching sessions with a Certified Diabetes Educator and support from a multidisciplinary virtual care team. Following the intervention, 11 participants and 6 virtual care team members were interviewed. Findings from this feasibility study highlighted a substantive need for responsive and whole-person care. Healthcare professionals, as part of the virtual care team, described the value of professional and clinical integration (e.g., addressing both physical and mental health needs in the same setting, and at the same time) and health coaching within a historically siloed and task-driven health system. Participants in this study described the ways that this program addressed their needs for service and support that was traditionally left unmet in previous health and social care interactions (Sherifali *et al.*, 2024). This included participants who were living with T2D for decades, those experiencing health inequities, and those living with serious mental illness and addictions:

*“I mean, I felt like I was able to talk about things with [Certified Diabetes Educator] that I did not have space to talk about before. I was able to identify what my problems were and what I needed, like resources or information or whatever, to make those changes. It never felt like we were doing anything really structured. It was like having a chat with a friend, but that friend had all this knowledge and this team of specialists to support her. It didn’t feel like she was following an itinerary or that she had specific steps to follow. It was organic and specific to me, and I think that’s why I was able to make some big changes for both my physical and mental health – changes that I have needed to make for many, many years. [Certified Diabetes Educator] helped me understand my health, like my eating needs and my addiction in ways that previous programs could not. I feel more confident in myself and my abilities”- TECC-DM Participant*

While findings from the TECC-DM feasibility study highlighted both the feasibility and the acceptability of this model of care, potential challenges to future implementations were also identified. This included issues with referrals, such as questions raised about how to refer people into the program, who would benefit from the TECC-DM program, how to refer those receiving TECC-DM to other community or health services, as well as communication challenges with both providing information to and receiving information from primary care:

*“I think one of the main challenges was that we haven’t ironed out completely how we link this person back to their family doctor?*

*How do we send information? And there was one point that I prepared a note for one of the participants to send a letter to their family doctor saying, ‘This came up. These are some of the suggestions we have,’ but I am not sure if that information was received, or what came of it.” – Virtual Care Team Member (Sherifali et al.,* *2024* *)*

The COVID-19 pandemic has contributed to a significant shift in how care is delivered. While healthcare has rapidly shifted to virtual and hybrid models of care delivery, there is further work to be done to uncover who these types of interventions may best support, how to identify individuals that may benefit from these programs, and how these interventions can be tailored, linked to, and delivered in healthcare settings. The purpose of this subsequent research project was to understand challenges to the delivery and uptake of personalized T2D care in primary and care settings by surveying primary care providers. This information is needed to inform integrated mental health support, and to refine the TECC-DM model of care.

## Methods

To address study objectives and inform the further refinement of the TECC-DM model of care, this study used a cross-sectional study design.

### Survey Design

Survey questions were developed collaboratively with input from the research team to reflect study aims. This included a review of questions for relevance and importance from a person with lived experience (PWLE) research team member. Further, survey questions were field tested by team members and three external healthcare professionals. These development steps, grounding in study objectives, input from a person with lived experience and expert review, support the content validity of the instrument within the context of the study’s quality improvement aims. The survey questions were developed to have respondents select responses to predefined questions that: (1) identified patient-level, inner-, and outer-practice setting factors that were perceived to challenge the delivery of personalized T2D care; (2) identified patient-related and healthcare system factors that were perceived to hinder the uptake of T2D self-management programs; and, (3) reflected on the potential application of the TECC-DM model of care, all in their setting and context. As the purpose of this survey was to further interpret TECC-DM feasibility findings and refine the model, a quality improvement approach was used, and no institutional ethics approval was required. Following a description of the survey and its purpose, survey respondents virtually consented to survey participation.

### Sampling

A convenience sampling approach was utilized, and the survey was distributed to all Smoking Treatment for Ontario Patients (STOP) providers via e-mail and promoted during STOP webinars. The STOP program is a global leader in smoking and vaping cessation treatment and education (Nicotine Dependence Service, 2025). STOP providers are multidisciplinary primary care providers and workers actively engaged in administering smoking cessation treatment in Ontario. There are over 1200 STOP providers from 412 healthcare sites across Ontario (Nicotine Dependence Service, 2025). This survey was distributed to all STOP provider sites as the TECC-DM feasibility study recruited from a database of current and previous STOP recipients, establishing a natural linkage. STOP providers were selected for this survey as they represent a large, multidisciplinary network of primary and community care providers actively engaged in behaviour change interventions, including chronic disease prevention and management. Given that the TECC-DM feasibility study recruited participants from a database of current and former STOP program users, engaging STOP providers created a natural alignment and allowed the research team to explore model applicability within familiar clinical and organizational settings.

The connection between cigarette smoking and T2D is well-established, with smoking exacerbating T2D-related complications and hindering effective disease management. Survey advertisement included an overview of the survey purpose and emphasized the focus on T2D care and management. All STOP providers were eligible to participate in the survey. All STOP providers were eligible to participate in the survey. No additional inclusion or exclusion criteria were applied.

### Analysis

Survey questions included a series of multi-select responses to questions about barriers and challenges, with opportunities for respondents to add “other” responses as free text. Further, respondents were asked to comment on their thoughts and ideas about the future application of TECC-DM via free text response boxes, which allowed for a more comprehensive exploration of respondent perspectives and experiences. Survey data were analyzed descriptively. This included summary statistics and frequency distribution of responses to predefined multi-select questions, as well as descriptive content analysis of free text responses. All free text responses were read and re-read, and like responses were grouped together and described, and illustrative quotes were selected. Analyses and interpretations were led by CW, with research team members and PWLE JF and AB contributing to this analysis and critical interpretation through independent analysis and iterative group discussions.

## Findings

85 unique respondents from 412 sites (20.6% response rate) completed the virtual survey between November and December 2023.

### Respondent Characteristics

Survey respondents (n = 85) represented a range of health professions, with the largest proportion being Registered Nurses (43.5%), Nurse Practitioners (10.5%), Pharmacists (9.4%), and Respiratory Therapists (9.4%). Survey respondents were employed in a range of primary and community-based settings, with the majority of respondents working in multidisciplinary health hubs, including family health teams (55.3%), community health centers (20%), or Aboriginal health centers (9.4%). As the survey was distributed to all STOP sites, survey responses were collected across Ontario. This included Central West Ontario (23.5%), Eastern Ontario (17.6%), Greater Toronto Area (16.5%), Southwest Ontario (15.3%), Northeast Ontario (10.6%), Central East Ontario (9.4%), and Northwest Ontario (7.1%). See Table 1 for respondent characteristics.

**Table 1.** Characteristics of survey respondents

Respondent Characteristics n = *85*		
Primary Role	n	Proportion (%)
Registered Nurse	37	43.53
Nurse Practitioner	9	10.59
Pharmacist	8	9.41
Respiratory Therapist	8	9.41
Social Worker	6	7.06
Health Promoter	5	5.88
Manager	3	3.53
Physician	3	3.53
Registered Dietitian	2	2.35
Registered Practical Nurse	2	2.35
Clinical Lab Assistant	1	1.18
Health Educator	1	1.18

Primary Setting	n	Proportion (%)
Family Health Team	47	55.29
Community Health Centre	17	20
Aboriginal Health Centre	8	9.41
Addiction and Mental Health Agency	4	4.71
Nurse-Practitioner Led Clinic	3	3.53
Public Health Unit	3	3.53
Medical Centre	1	1.18
Solo Primary Care Practice	1	1.18
Regional Diabetes Education Program	1	1.18
Long-Term Care Facility	0	0

Primary Location	n	Proportion (%)
Central West Ontario (e.g., Hamilton, Kitchener, Guelph)	20	23.53
Eastern Ontario (e.g., Kingston, Ottawa)	15	17.65
Greater Toronto Area	14	16.47
Southwest Ontario (e.g., London, Windsor)	13	15.29
Northeast Ontario (e.g., Sudbury, Timmins)	9	10.59
Central East Ontario (e.g., Peterborough, Barrie)	8	9.41
Northwest Ontario (e.g. Thunder Bay, Sioux Lookout)	6	7.06

### Personalized T2D Care Delivery

Survey respondents were asked to reflect on patient-level and inner- and outer-practice setting factors that challenge the delivery of personalized T2D in their settings (see Table 2). These questions were *select all that apply* and included an option for respondents to add their ideas as free text, as such only the frequency of responses are described.

**Table 2.** Factors that hinder the delivery of personalized T2D care

Challenges to the Delivery of Personalized T2D Care	
Patient-Level Challenges	n (select all that apply)
Limited or lack of engagement	49
Limited or lack of interest	41
There are no patient level challenges	18
Other (see examples in the description)	17
Illustrative Quotes: “More urgent needs like housing, food access, violence and unstable mental health issues.” “Health inequities affecting social determinants of health, especially including chronic stress, income and food insecurity - many competing priorities trump having capacity to take on diabetes care.”	

Inner-Practice Challenges	n (select all that apply)
Limited resources (e.g., staffing)	45
Time constraints	45
Administrative burden	20
Limited workflow for chronic disease management	17
Limited ability to prioritize this care	15
Limited ability to screen for mental health challenges	13
Limited access to data or ability to collect data	13
Limited knowledge or access to knowledge (e.g., up-to-date guidelines)	10
There are no inner-practice setting challenges	19
Other (see examples in the description)	4
Illustrative Quotes: “Staff absences impact continuity of care for each client.” “Diabetes care is complex. We don’t always have enough time to do all that we need to.” “Short visit - limited ability for patient to get all the education and information they need”	

Outer-Practice Challenges	n (select all that apply)
Issues with access (e.g., to specialized diabetes or mental health teams)	46
Waitlists	40
There are no outer-practice setting challenges	16
Other (see examples in the description)	9
Illustrative Quotes: “Patients see other providers and they receive inconsistent or conflicting advice.” “Silos within health care settings and with social services and health care.”	

#### Patient-Level

At the patient-level, survey respondents identified that limited or lack of engagement (n = 49) and limited or lack of interest (n = 41) most challenged delivery of this care. Other responses emphasized the health inequities that patients lived with, and offered further explanation specific to the limited or lack of engagement responses. These included “more urgent needs like housing, food access, violence and unstable mental health issues” or “chronic stress, income insecurity”. One respondent shared that while diabetes care was important, there were “many competing priorities [that] trump having capacity to take on diabetes care” for some patients they supported.

#### Inner-Practice

Inner-practice setting challenges most commonly reported included limited resources (n = 45), time constraints (n=45), administrative burden (n=20), limited workflow for chronic disease management (n = 17), and a limited ability to prioritize this care (n = 15). Some respondents (n = 19) felt that no inner-practice settings were hindering this care delivery.

#### Outer-Practice

From an outer-practice setting perspective, survey respondents identified issues with access, such as those with limited means to specialized diabetes or mental health teams (n = 46), and waitlists (n = 40) were significant challenges to T2D care. Other responses identified existing barriers at the local level, including staff absences.

### Uptake of T2D Programs

Survey respondents were then asked to consider patient-related and healthcare system factors that may hinder the uptake of T2D self-management programs (see Table 3).

**Table 3.** Factors that hinder the uptake of personalized T2D care

Challenges that Hinder the Uptake of Personalized T2D Care	
Patient-Related Factors	n (select all that apply)
Reluctance to change lifestyle or behaviors	64
Financial barriers	59
Complexity or other comorbidities	53
Reluctance to track health (e.g., blood glucose, carbohydrate intake)	50
Limited awareness	42
Reluctance to take medication	40
Limited social support	37
Fear (e.g., of unknown, complications)	26
Cultural beliefs or practices	23
There are no patient-related factors	3
Other (see examples in the description)	7
Illustrative Quotes: “Access to reliable transportation make it difficult for some patients.” “Not everyone has access to what is recommended, like healthy foods.”	

Healthcare System Factors	n (select all that apply)
Challenges in integrating self-management into primary care visits	39
Limited accessibility or availability of diabetes programs	34
Limited healthcare provider engagement	29
Challenges in sharing data	28
Unclear evidence for available programs	10
There are no challenges with uptake of these programs	7
Other (see examples in the description)	6
Illustrative Quotes: “Highly complex clients with multiple comorbidities needing to access multiple healthcare providers but we are understaffed and have a high workload.” “People have to keep telling their story every time they access a service.”	

#### Patient Factors

Specific to patient factors, survey respondents identified a reluctance to change lifestyle or behaviours (n = 64), financial barriers (n = 59), complexity or other comorbidities (n = 53), reluctance to track health, such as blood glucose (n = 50), limited awareness (n = 42), and reluctance to take medication (n = 40) were all factors that were perceived to contribute to hindered uptake of these programs.

#### System Factors

Healthcare system factors included challenges to integrating self-management into primary care visits (45.9%), limited accessibility or availability of diabetes programs (40%), limited healthcare engagement (34.1%), and challenges in sharing data (32.9%). Other responses expanded upon some of the inequities described above. These included comments about patients “feeling overwhelmed” with their care, and how patients may know what they need to do, not all of them have “access to recommended foods”.

### Application of TECC-DM

Following these questions, respondents were then asked to reflect on the application of the TECC-DM model of care. This included opportunities to contribute free-text responses to questions about who would benefit from a program like TECC-DM, the changes or adaptations that would be needed to increase program accessibility, and any community assets (e.g., organizations or resources) that would support this work. Of the 85 survey respondents, 57 respondents (67.1%) completed these additional questions in the survey.

Survey respondents (n = 57) identified a range of responses to those they thought would benefit from the integrated and collaborative care offered in TECC-DM. This included individuals living with chronic conditions, such as diabetes, but also chronic obstructive pulmonary disease or other metabolic conditions, as well as those with “complex health needs” and multimorbidity. Respondents described programs like TECC-DM as needed for those who “prefer consistent, ongoing support and guidance in managing their diabetes”, “enjoy being a part of their care”, or those who were “newly diagnosed” as beneficiaries of this structured programming. Survey respondents described the need for integrated physical and mental health care, citing the benefits of these healthcare providers being aware of the care and support being provided by others:

*“The patients would benefit having their health care providers and their mental health workers all on the same page for their complete care.”*

*– Survey respondent*

Respondents also identified that those who were not connected to a primary care provider, those who lived in rural areas, and those who experienced mobility (e.g., functional limitations) and transportation challenges would likely benefit from TECC-DM. Interestingly, survey respondents also identified who they thought the program would not benefit, or who, if engaged in the program would need additional support. This included discussions of the limitations of virtual care for those who live in rural areas (e.g., limited access to adequate internet for web-conferencing), as well as a need to consider that individuals who are “engaged and self-motivated” in their diabetes care would fare better as TECC-DM patients would need to “make time for the program.” This is particularly challenging when many respondents identified that younger individuals living with T2D would most benefit, but that the program’s “weekly follow-ups could be a burden given that these are the people who also work full-time and have young families.”

Suggested changes or adaptations for future applications of the TECC-DM model included recommendations for virtual care team membership, including the addition of nurse practitioners, pharmacists, or dietitians, and the need to partner with local community organizations and resources, such as libraries, to ensure access to devices and space. Respondents felt that these partnerships could assist in overcoming challenges not only with access, but also to support specific populations, like older adults and newcomers. Referrals to the program from primary care were identified as a potential challenge, and respondents identified a need for “an easy referral process” where there was “ease of transfer of patient information.” This extended to a need for the program to facilitate opportunities for primary care team members to attend virtual care team meetings and contribute to care. One respondent asked:

*“How many family physicians attended the [virtual care team] meetings? Providing an invite and actually being able to attend are completely different things.” – Survey respondent*

Another respondent questioned how a program like TECC-DM could support existing health human resource challenges in primary care:

*“The shortage of regulated professionals in the health care system is the biggest problem in making this an accessible program. The current demand on our health care providers would not leave them enough time to attend [these meetings].” - Survey respondent*

Lastly, survey respondents provided recommendations on community-based organizations in their individual regions that could be involved in a program like TECC-DM. These responses largely identified diabetes-specific resources, but other more social-service type suggestions were posed as well. These included a need to work with local food banks or visiting programs to ensure that resource needs were met for everyone.

## Discussion

Findings from this research have identified challenges in delivering personalized T2D care and in the uptake of integrated self-management programs in primary care. These identified challenges at the patient, setting, and system levels underscore the complexity associated with integrating such programs into routine care. Moreover, these challenges confirm that for program adoption to succeed, comprehensive and multifaceted strategies that address these challenges are needed. Findings identified in this survey are like other research that has explored provider’s thoughts on integrating programs or interventions in primary care settings. For example, in research that has explored the integration of brief alcohol interventions with concurrent tobacco addiction treatment in primary care, providers similarly described a lack of time and a lack of resources to support change (e.g., training, education) as barriers to deliver and uptake (Minian *et al.*, 2021). Key findings from this survey will guide our team as we look to mobilize feasibility findings and refine the TECC-DM model further. These include an opportunity to tailor the model to meet person-centred needs, address mental health support, and build strong partnerships.

### Tailoring the Model to Individual Needs

Respondent insights regarding who would benefit from the TECC-DM model were contrasting. Many responses emphasized an opportunity for TECC-DM to support those living with multimorbidity, complexity, or with challenges to access (e.g., transportation, language, socioeconomics). Conversely, others perceived TECC-DM as a model that would benefit “anyone living with diabetes,” with no immediate patterns in these responses concerning role or setting. On the surface, these findings could be interpreted as disparate, however alternatively these findings present an opportunity to consider the potential inclusive and comprehensive nature of TECC-DM. Perceived to be capable of adequately responding to or addressing complexity, while also being described as benefiting those without this complexity, signals an ability of models of care like TECC-DM to provide personalized care to extend beyond traditional boundaries and encompass individuals with varying levels of complexity, accessibility, and motivation.

The need for personalized diabetes care is paramount to improving health outcomes and enhancing the overall quality of life for individuals living with T2D (Robinson *et al.*, 2023, Sherifali *et al.*, 2018). By tailoring care plans to each individual’s unique needs, preferences, and circumstances, personalized diabetes care can optimize treatment effectiveness, promote patient engagement and adherence, and ultimately lead to improved glycemic targets and reduction in the risks of diabetes-related complications (Siminerio *et al.*, 2013, Rutten *et al.*, 2020, Sherifali *et al.*, 2018). Additionally, personalized care acknowledges the diverse array of factors that influence diabetes management, including socioeconomic status, cultural background, health literacy, and psychosocial factors (Government of Canada, 2022, Nam *et al.*, 2011, Gonzalez *et al.*, 2016). Through personalized care, individuals can receive tailored support and resources that align with their goals and priorities, fostering a sense of empowerment and self-efficacy in managing their condition. In the context of TECC-DM, this personalization should include flexibility in the frequency and intensity of the health coach contact, the expertise at the virtual care team table, and the focus of the support (e.g., resources) and planning (e.g., referrals) provided. Through this personalization, models like TECC-DM can prioritize individual needs and preferences while improving health outcomes and enhancing overall well-being.

To support sustainable implementation of integrated care models like TECC-DM, strategies must account for both the clinical and operational realities of primary care, including workload, health human resource constraints, and the need for workflow alignment. Emerging implementation science frameworks, such as the Consolidated Framework for Implementation Research (CFIR), underscore the importance of tailoring interventions to local context, engaging stakeholders early, and addressing inner and outer setting factors that influence uptake and fidelity (Damschroder et al., 2009).

### Addressing Mental Health Support

Survey findings revealed a notable absence of the explicit discussion of mental health, including current practices specific to mental health assessments and treatment, or the ways that mental health may already be integrated into diabetes care and support. Also, survey respondents largely identified diabetes-specific resources, with little reference to mental health resources available in their communities upon question of potential community resources, collaborations, or relevant connections. The absence of these findings is noteworthy as the survey described the model as an integrated diabetes and mental health intervention, shared findings from the feasibility study specific to mental health needs and gaps, and asked questions about this need for integration in practice. This gap can be interpreted to represent a larger symptom of a fragmented healthcare system, where the integration of mental health care into chronic condition management is not prioritized, incentivized, or sufficiently resourced, leading to limited support for and awareness of options to deliver comprehensive care. Further, this gap underscores an urgent need to emphasize mental health support within T2D care models, recognizing and acting upon the interdependence of physical and mental well-being in achieving optimal health outcomes.

To bridge this gap, strategies to integrate mental health screening, assessment, and intervention into diabetes care programs are needed. This integration acknowledges the complex interplay between diabetes and mental health, ensuring that individuals receive the support they need to manage both aspects of their health effectively (Robinson *et al.*, 2023, Cimo and Dewa, 2019). Specific to TECC-DM, this could include further training health coaches to communicate with individuals about their mental health and identify potential challenges in the early stages of care. In addressing mental health challenges proactively, the health coach could help mitigate the negative impact of these challenges on diabetes management (Gonzalez *et al.*, 2008), ultimately improving the ability to achieve glycemic targets, reducing the risk for diabetes-related complications, and enhancing quality of life (Perrin *et al.*, 2017, Owens-Gary *et al.*, 2018). Further, the inclusion of mental health specialists within the TECC-DM virtual care team, including psychologists, allied health team members, and psychiatrists, may enhance the skill and experience of the health coach, and overcome what may be limited or missing in the community.

### Building Strong Partnerships

Respondent suggestions for change and adaptation to enhance the TECC-DM model highlight the importance of collaborative and community-driven implementation and scale-up. Recommendations for partnerships with community organizations, including libraries, food banks, and home care, to address access issues and support specific populations demonstrate the potential for synergy between traditional healthcare providers and community spaces with local and social resources. Within increasingly strained systems, these collaborations are not only beneficial for individuals and communities, but are necessary to foster resilience and sustainability in health and social care delivery.

Collaborative health interventions that focus on care coordination to support chronic condition management continue to largely intervene at the level of the individual patient despite a considerable evidence base identifying broad patient, care team, organization, system, and policy-level factors that influence this type of coordination (Weaver *et al.*, 2018, Peterson *et al.*, 2019, Albertson *et al.*, 2022). Care for those living with chronic conditions, including diabetes and mental health challenges often involves a network of teams and groups working toward shared goals and across boundaries (Weaver *et al.*, 2018, Albertson *et al.*, 2022). An ability to tailor the TECC-DM model, to include the flexibility to engage with existing community assets, referrals to external resources, and collaboration with diverse stakeholders, can all help to address these broad factors and interdependencies that present within and between those caring for mutually shared individuals. These opportunities could support the implementation of the model into routine practice, underscore the importance of seamless integration with existing structures, and represent the ways that innovation can be sustained through judicious application and continuous improvement.

## Practical implications

The findings from this study reveal actionable strategies for applying the TECC-DM model in primary care settings. These practical implications offer guidance for future implementation efforts.

### Integrating mental health support

The TECC-DM model must prioritize the integration of mental health support into diabetes care. This includes embedding mental health screening and assessment into routine workflows, training health coaches to identify early signs of mental health challenges, and incorporating mental health specialists (e.g., psychologists, social workers, or psychiatrists) into the virtual care team. By addressing mental health challenges alongside diabetes management, the program can improve patient outcomes and reduce the burden on fragmented care systems.

### Strengthening community partnerships

Collaboration with community-based organizations is essential to addressing structural barriers and enhancing accessibility. Partnerships with resources such as libraries (to provide access to internet and technology), food banks (to address nutritional needs), and local transportation services can mitigate barriers that patients face in engaging with care. Leveraging these resources ensures that the TECC-DM model is responsive to the social determinants of health.

### Tailoring the model to local needs

Flexibility in the design and delivery of the TECC-DM model is critical for its success across diverse primary care settings. Recommendations include enabling care teams to adjust the frequency and intensity of interactions based on patient needs, integrating additional providers such as nurse practitioners or pharmacists into the virtual care team, and creating adaptable workflows to align with local practice settings. Tailoring the program in this way ensures its relevance and feasibility in varied contexts.

## Study limitations

This study has several limitations that should be considered when interpreting its findings. First, the sample was restricted to healthcare providers in Ontario, which may limit the applicability of the results to other regions with differing healthcare systems or practices. Second, the use of convenience sampling may introduce sampling bias, as participants who opted into the survey might differ systematically from those who did not, potentially influencing the findings. Finally, the findings may not be generalizable beyond the Ontario region due to the specific jurisdictional healthcare context and programmatic structures in place. Additionally, the survey response rate of 20.6% introduces the possibility of non-response bias, whereby the perspectives of providers who chose not to participate may differ in meaningful ways from those who did, potentially limiting the representativeness of the findings. Future studies should aim to include a more geographically diverse sample and employ alternative sampling strategies to enhance the representativeness and applicability of the findings.

## Conclusion

With primary and community care systems overwhelmed by increasing demands and limited resources, the needs of individuals living with T2D and mental health challenges may be inadequately addressed, contributing to gaps in support and care. An understanding of the ways in which these individuals access – or fail to access – treatment, including the barriers and challenges to integrated models of care in these settings, is needed to achieve optimal, whole-person care. Study findings identified will inform the refinement of the TECC-DM model, including further testing of the model and its effectiveness. By addressing the identified challenges and leveraging collaborative partnerships, TECC-DM holds promise in improving health outcomes and enhancing the overall quality of life for individuals living with T2D and mental health challenges.
